# Clinical, hematological and some biochemical alterations during diarrhea in Friesian calves naturally infected with *E. coli* and *Salmonella*

**DOI:** 10.1186/s43088-022-00309-w

**Published:** 2022-10-04

**Authors:** Ahmed Shehta, Heba El-Zahar, AbdelKereem Mansour, Basma Mustafa, Tarek Shety

**Affiliations:** Internal Medicine, Department of Animal Medicine, Faculty of Veterinary Medicine, Zagazig University, Zagazig, 44511 Ash Sharqia Egypt

**Keywords:** Bovine calves, Diarrhea, Hemato-biochemical, Vit D_3_, Cardiac biomarkers

## Abstract

**Background:**

This study aimed to assess the clinical and hemato-biochemical changes associated with diarrhea in *E. coli* and *Salmonella* pathogens in Friesian bovine calves less than one month old as well as to examine the relationship between Vit D_3_ and cardiac biomarkers.

**Results:**

The study was carried out on 43 Friesian calves from a private farm in *Ash Sharqia Governorate*, 33 diarrheic calves aged 1–14 days with an average body weight 43.7 ± 1.2 kg, and 10 apparently healthy calves were kept as a control group to investigate the clinical and hemato-biochemical profiles. *E. coli* and *Salmonella* pathogens were isolated from the diarrheic calves where 76% were *E. coli* and 24% were* Salmonella*. Diarrheic calves showed signs of anorexia, weakness, dullness, staggering gait, pale mucous membranes with sunken eyes and dehydration. Analysis of clinical and hemato-biochemical profile of the diarrheic calves revealed significant increase in body temperature, respiratory rate, heart rate, white blood cells, neutrophils, serum potassium, urea, creatinine, ALT, AST, cardiac NT-proBNP and cardiac troponin I with significant decrease in hemoglobin, packed cell volume, erythrocytes, serum Vit D_3_, sodium, glucose, total protein and albumin concentrations.

**Conclusions:**

It could be concluded that bacterial diarrhea has a severe negative impact on the clinical and hemato-biochemical profile of the neonatal calves with diarrhea. Serum cardiac biomarkers such as cardiac troponin I (cTn-I) and NT-proBNP were significantly higher in neonatal calves with diarrhea, and there is an important relationship between cardiac biomarkers and vitamin D levels.

## Background

One of the most serious problems in cattle breeding is calf diarrhea, which is the major cause of neonatal mortality in both dairy and beef calves less than one month old, as well as financial losses due to medical treatment costs, growth retardation and even death [1, 2].

A variety of factors can contribute to calf diarrhea. In most cases, non-infectious causes as inadequate nutrition, environmental conditions, management practices and infectious causes such as bacteria, viruses and protozoa [3]. Cryptosporidium spp., bovine rotavirus, bovine coronavirus, enterotoxigenic *E. coli* and *Giardia* spp. are the most common endemic microorganisms implicated in neonatal calf diarrhea [4]. These infectious agents, alone or in combination with other infections, can cause diarrhea in calves [1, 5–7]. Many factors contribute in the occurrence of diarrhea in newborn calves, including the number of animals in the farm, colostrum intake, vaccination program and umbilical cord problems. In addition, farm type, shelter structure, season, poor cleaning and inappropriate disinfection in shelters and age are all risk factors that influence the disease emergence and severity [8].

Regardless of the cause, diarrhea can result in dehydration, electrolyte imbalance, metabolic acidosis and hypovolemia. Hypovolemia results in kidney failure and heart block due to hyperkalemia and septicemia due to secondary bacterial overgrowth in the small intestine. The kidney reduces urine production to compensate for the increased fluid losses caused by diarrhea [9–11].

Early detection is expected to reduce losses from existing cases and prevent new ones from occurring. As a result, having a quantitative indicator for early detection of symptoms associated with these complications would be extremely beneficial to the success of treatment and would reduce mortality [12, 13].

Calves suffering from diarrhea are dehydrated, have a decreased appetite and have difficulty in standing [14]. Calves suffering from severe diarrhea develop ataxia, acidemia, bacteremia, arrhythmia and hypovolemia, all of which can lead to death. Veterinary assessment of diarrheal calves is typically based solely on clinical examination and determining the degree of dehydration in diarrheic calves [15].

Evaluation of 25 (OH) D_3_ levels is related to different diseases and operative conditions [16, 17]. It was observed that Vit D can limit the occurrence of diarrhea and increase the resistance to diarrhea [18, 19]. In addition, Vit D has also been reported to protect against intestinal surface infections and prevent leaky gut syndrome and regulates the inflammatory response and activates immune cells in this process [20]. Cardiovascular biomarkers such as cardiac troponin I (cTn-I) and NT-proBNP aid in the early detection of cardiac disorders [19]. The relationships between cardiac biomarkers and vitamin D levels in human and animal medicine have been demonstrated by many researchers and this relationship is important in terms of cardiometabolic diseases [16, 19, 21]. However, studies evaluating the changes between inflammatory biomarkers and vitamin D levels in different diseases are gaining importance recently [22, 23].

The aim of this study was to assess the clinical and hemato-biochemical changes associated with bacterial diarrhea in Friesian calves less than one month old. In addition, to determine the plasma 25 (OH) D_3_ concentrations in calves with diarrhea symptoms and to examine the relationship between 25 (OH) D_3_ and cardiac biomarkers.

## Materials and methods

### Animals

Forty-three newly born bovine Friesian calves of both sexes in a private farm in *Ash Sharqia Governorate* were used in the present study, of which 33 calves with neonatal calf diarrhea (age range 1–14 days old) and 10 apparently healthy calves were kept as a control group in the same age range. The control calves showed no history of previous illness. In all calves and immediately after birth, the navel was disinfected daily for the first three to four days of life to prevent infection, all calves received 2–3 l of colostrum by bottle-feeding system within the first few hours after birth and then housed individually in a single pen. Every day, calves were fed cow's milk (twice a day) at about 10% of their body weight. Because this study did not include any experimental work, approval from the Zagazig University Institutional Animal Care and Use Committee (ZU-IACUC) was not required.

### Clinical examination

A thorough clinical examination was performed for all calves in the study either clinically healthy or diarrheic. Body temperature, respiration and heart rate were recorded using the methods described by Constable et al. [24]. Fecal samples from calves without or with diarrhea were examined for consistency, color, odor and presence of foreign elements.

### Blood sampling and analysis

Blood samples were collected for measuring the hematological and biochemical parameters before starting therapy, two venous blood samples were collected from jugular vein. The first sample is a heparinized whole blood sample for measuring complete blood count, including hematocrit (%), hemoglobin (%), erythrocyte count (× 10^6^/µl) and total leukocytic count(× 10^3^/µl) using automated cell counter (HA-Vet Hematology Analyzer®, Clindiag Systems B.V.B.A, Belgium) according to the standard methods.

The second sample put in plain tube without anticoagulant for collecting serum after centrifugation of the sample for 10 min at 3000 rpm, serum samples were collected and then kept frozen at − 20 °C until further analysis. Serum samples were used for analysis of serum aspartate aminotransferase (AST) and alanine aminotransferase (ALT) and concentrations of total protein, albumin, serum creatinine, blood urea nitrogen, glucose, Na, K and Cl, using Beckman AU5800 analyzer (Beckman Coulter, California, USA). Serum vitamin D_3_concentration was measured using Siemens ADVIA Centaur XP analyzer (Siemens Medical Solutions, Malvern, USA). In addition, the concentration of serum cTn-I was determined with a commercial kit (Card-I-kit Combo Test; Aboa Tech). While the concentration of (NT-proBNP) was measured using sandwich enzyme immunoassay tests (ELISA).

### Fecal samples

Two fecal samples were taken in a clean dry plastic pack. The first sample for parasitological examination to detect gastrointestinal parasites according to method described by Zajac et al. [25]. The second sample for bacteriological analysis using one gm of each fecal sample was diluted in 2 ml of sterile saline then double fold serially diluted; 100 microns from each dilution was inoculated in the culture media. Samples were cultured and identified according to Quinn et al. [26]. For isolation of *Salmonella strains*, a swap from the diluted specimens was inoculated into tetrathionate broth with overnight incubation at 37 °C, then subculture on xylose lysine deoxycholate and *Salmonella–Shigella* agar media and incubated at 37 °C for 18–24 h. Suspected grown isolates were subjected to biochemical testing according to Collee et al. [27].

For isolation of *E. coli strains*, a swap from the diluted specimens was inoculated into MacConkey’s agar and incubated at 37 °C for 18–24 h. Lactose fermenter (pink) colonies were subculture on Eosin Methylene blue agar and confirmed as *E. coli* using the standard biochemical tests according to Collee et al. [27].

### Statistical analysis

All data were statistically analyzed using SPSS Statistics®22.0 (Version 22.0, Armonk, NY: IBM Corp). The data were tested for normal distribution using *Shapiro Wilks W Test* and were found normally distributed. The obtained results were analyzed using T-test, all data are listed as mean ± SE. Differences between parameters were tested for significance at *P* < 0.05. Pearson correlation was used for estimating the relationship between the concentration of cardiac inflammatory markers (cardiac ProBNP and cTn-I) and Vit D_3_ in neonatal diarrheic calves.

## Results

### Clinical findings

The most common clinical findings in calves during the diarrheal stage, based on clinical examination are summarized in Table 1, anorexia (94%), staggering movement (76%), sunken eyes (82%), diarrhea (100%), mild dehydration (64%), moderate dehydration (12%), dullness and pale mucous membranes were also recorded. The degree of dehydration was measured based on the skin fold test time and eye condition; calves with mild degree of dehydration have slow skin fold visible for less than 2 s with non-sunken eye. In moderate degree of dehydration, calves showed slow skin fold visible for more than 2 s (2–4 s) with the eye barely sunken. The color of diarrheal stools varies from pale to dark yellow, greenish and milky white, with flecks of mucus or blood visible in the majority of diarrheic calves. As well, the remaining 10 calves (control group) showed no signs of diarrhea or observable abnormalities based on clinical examination findings.

**Table 1:** Frequencies of the clinical signs in diarrheic and healthy calves included in the present study, the data are presented as absolute number and their percentages [n (%)]

	Healthy calves (*n* = 10)	Diarrheic calves (*n* = 33)
Anorexia	None	31 (94%)
Staggering movement	None	25 (76%)
Sunken eyes	None	27 (82%)
Diarrhea	None	33 (100%)
Dehydration		
Nil	None	8 (24%)
Mild	None	21 (64%)
Moderate	None	4 (12%)

There was a significant (*p* < 0.05) increase in the mean rectal temperature, the mean respiration and heart rate (Table 2).

**Table 2:** Clinical examination parameters in diarrheic and healthy calves included in the present study

Parameters	Healthy calves (*n* = 10)	Diarrheic calves (*n* = 33)	Reference values
Body weight (Kg)	43.7 ± 1.2^a^	37.79 ± 1.8^b^	–
Rectal temperature (°C)	38.6 ± 0.1^b^	39.7 ± 0.2^a^	38.5–39.5 [24]
Respiratory rate (breaths/min)	25.48 ± 1.27^b^	34.12 ± 2.17^a^	20–50 [41]
Heart rate (beats/min)	98.5 ± 2.5^b^	123 ± 3.31^a^	126 ± 4 [36]

The data are presented as mean ± SE

^a,b^Means within the same row with different superscripts differ significantly (*P* < 0.05)

### Hematological and biochemical findings

The mean values of hematological indices in diarrheic calves are summarized in Table 3. Complete blood count showed significant reduction in total erythrocyte count (7.29 ± 0.17 × 10^6^/μl) and hemoglobin (8.8 ± 0.16 g/dl) compared to the control group measurements (8.71 ± 0.15 × 10^6^/μl and 11.44 ± 0.33 g/dl, respectively). While, there was a significant increase in the total white blood cell count (16.2 ± 0.74 × 10^3^/µl), neutrophils (72.02 ± 0.56%) and HCT (37.78 ± 1.16%) compared to the results of the control group measurements (8.93 ± 0.27 × 10^3^/µl, 58.1 ± 2.4% and 31.47 ± 0.35%, respectively).

**Table 3:** Hematological and biochemical parameters in diarrheic and healthy calves included in the present study

Parameters	Healthy calves (*n* = 10)	Diarrheic calves (*n* = 33)	Reference values
Hemoglobin (g/dl)	11.44 ± 0.33^a^	8.8 ± 0.16^b^	Navetat et al. [9]
Hematocrit (%)	31.47 ± 0.35^b^	37.78 ± 1.16^a^	
Erythrocytes (× 10^6^/µl)	8.71 ± 0.15^a^	7.29 ± 0.17^b^	
Leukocytes (× 10^3^/µl)	8.93 ± 0.27^b^	16.2 ± 0.74^a^	
Neutrophils (%)	58.1 ± 2.4^b^	72.02 ± 0.56^a^	
Total proteins (g/dl)	7.08 s ± 0.09^a^	6.28 ± 0.28^b^	
Albumin (g/dl)	3.85 ± 0.17^a^	3.17 ± 0.02^b^	
Globulin (g/dl)	3.23 ± 0.13^a^	3.1 ± 0.27^a^	
BUN (mg/dl)	26.03 ± 0.96^b^	45.21 ± 0.32^a^	
Creatinine (mg/dl)	0.49 ± 0.05^b^	1.51 ± 0.21^a^	
ALT (u/l)	61.24 ± 1.41^b^	77.27 ± 2.07^a^	
AST (u/l)	84.18 ± 0.94^b^	117.14 ± 3.46^a^	
Glucose (mg/dl)	106.71 ± 2.7^a^	79.23 ± 1.34^b^	
Na^+^ (mmol/l)	135.18 ± 0.78^a^	123.04 ± 1.05^b^	
K^+^ (mmol/l)	4.47 ± 0.06^b^	5.26 ± 0.15^a^	
Cl^+^ (mmol/l)	95.2 ± 1.1^a^	76.84 ± 0.7^b^	

The data are presented as mean ± SE

^a,b^Means within the same row with different superscripts differ significantly (*P* < 0.05)

The concentrations of serum Na and Cl levels were significantly (*p* < 0.05) decreased while serum K level was significantly (*p* < 0.05) increased in the diarrheic calves compared to healthy calves (Table 3).

In addition, there was a significant (*p* < 0.05) increase in blood urea nitrogen, creatinine, ALT and AST levels, while total protein, serum albumin and glucose concentrations showed significant (*p* < 0.05) decreases in the diarrheic calves compared to healthy calves (Table 3).

The results of serum NT-proBNP and cTn-I levels were significantly (*p* < 0.05) increased, while vitamin D_3_ concentrations showed significant (*p* < 0.05) decreases in the diarrheic calves compared to healthy calves (Table 4).

**Table 4:** Serum cardiac biomarkers concentration in diarrheic and healthy calves included in the present study

Parameters	Healthy calves (*n* = 10)	Diarrheic calves (*n* = 33)	Reference values
Vit D_3_ (ng/ml)	64.37 ± 0.72^a^	29.97 ± 3.3^b^	Navetat et al. [9]
NT-proBNP (pg/ml)	31.5 ± 2.42^b^	112.7 ± 6.11^a^	
cTn-I (ng/ml)	0.21 ± 0.02^b^	0.39 ± 0.01^a^	

The data are presented as mean ± SE

^a,b^Means within the same column with different superscripts differ significantly (*P* < 0.05)

**Fig. 1:**
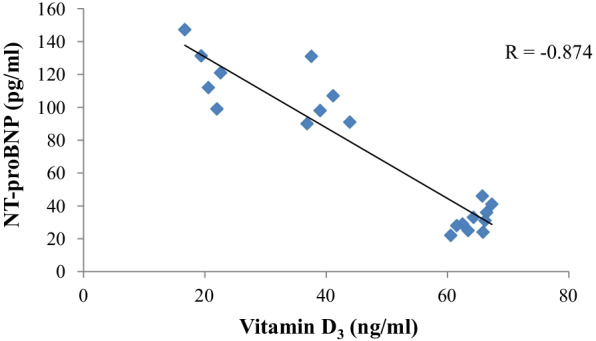
The correlation between serum vitamin D_3_ and NT-proBNP in 33 diarrheic calves. The graph illustrates a strong negative correlation (*r* = −0.874) between vitamin D_3_ (ng/ml) and NT-proBNP (pg/ml), *r* = Pearson correlation coefficient

**Fig. 2:**
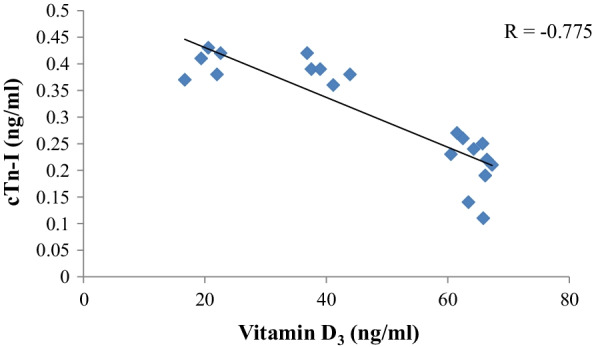
The correlation between serum vitamin D_3_ and cTn-I in 33 diarrheic calves. The graph illustrates a strong negative correlation (*r* = −0.775) between vitamin D_3_ (ng/ml) and cTn-I (ng/ml), *r* = Pearson correlation coefficient

The relationship between the serum cardiac biomarkers NT-proBNP, cTn-I and the vitamin D_3_ concentrations, showed very strong negative correlation (*r* = −0.874, *r* = −0.775, respectively) (Figs. 1, 2).

### Fecal analysis and Bacteriological findings

Fecal samples were examined using routine fecal examination, it was negative for fecal parasites. Pathogenic *E. coli* was isolated from the fecal samples from 25 diarrheic calves which represented 76% of the diarrheic calves (Table 5) with a bacterial count (4.5 × 10^7^ CFU/gm) (Table 6), while *Salmonella* spp. Was isolated from the fecal samples from 8 diarrheic calves which represented 24% of the diarrheic calves (Table 5).

**Table 5:** Bacteriological examination of fecal samples from 33 diarrheic bovine calves

Bacterial isolate	Positive samples (*n*)	Isolation % in diarrheic calves
Pathogenic *E. coli*	25	76
*Salmonella* spp.	8	24
Other pathogens	–	–

**Table 6:** Pathogenic *E. coli* bacterial count of fecal samples from healthy and diarrheic bovine calves

Animal group	CFU/g
Healthy calves (*n* = 10)	< 100
Diarrheic calves (*n* = 33)	4.5 × 10^7^

## Discussion

For calves younger than one month, neonatal diarrhea is a major cause of illness and death. Regardless of the underlying pathogens or pathophysiologic mechanisms, diarrhea can result in death from dehydration, acidemia, hyperkalemia and impaired cardiovascular and renal function [28].

It can result in neonatal mortality in dairy and beef calves less than one month old, as well as financial losses due to medical treatment costs, growth retardation and even death [1, 2, 29, 30]. The most common clinical observations in calves during diarrhea were anorexia (94%), staggering movement (76%), sunken eyes (82%), diarrhea (100%), mild dehydration (64%), moderate dehydration (12%), dullness and pale mucous membranes. The color of diarrheal stools varies from pale to dark yellow, greenish and milky white, with flecks of mucus or blood visible in the majority of diarrheic calves that was in accordance to Ghanem et al. [31], Constable et al. [24] and Özkan et al. [32]. The obtained results were also in agreement with previous findings reported by El-Seadawy et al. [33] and Torche et al. [34], who observed that the most clinical signs include the presence of soft feces watery in consistency and its color ranged from greenish, yellowish or blackish according to the causative agent, dullness, pale mucous membranes with sunken eye due to excessive water and electrolytes loss.

As infectious diarrhea is a common condition affecting newly born calves, bacteriological examination in the current study revealed that 76% of the diarrheic calves had pathogenic *E. coli* and a 24% had *Salmonella species* isolated from the fecal samples. *Salmonella species* and *E. coli* are known as the most common pathogens identified in diarrheic calves. These results were consistent with previous findings reported by El-Seedy et al. [35] and El-Seadawy et al. [33].

There was a significant increase in the mean values of rectal body temperature, respiration and heart rates in calves suffered from diarrhea. This observation coincides with previous reports [36, 37]. In fact, it appears that a compensatory polypnea in response to acidosis to eliminate the excess of CO_2_ in order to reach normal values of pH and tachycardia compensates the hypovolemia due to dehydration during diarrhea [38].

Furthermore, rectal temperature could be influenced by the duration of the diarrhea and the severity of dehydration, first there is an increase in temperature in early stage of diarrhea (39 to 40.5 °C) and then, it decreased in severe cases of diarrhea (36–38 °C) [34].

The mean values of hematological indices in diarrheic calves showed significant reduction in total erythrocyte count and hemoglobin. While, there was a significant increase in the total white blood cell count, neutrophils and HCT compared to the results of the control group. The increase in WBCs and neutrophilia in calves suffered from diarrhea than apparently healthy group is due to the infection by pathogenic *E. coli* and *Salmonella* spp. The significant increase in the hematocrit was observed during scours in other studies [36, 37, 39, 40]. Hematocrit provides important information about the overall blood volume; in addition, it tended to be higher at birth and then decreased with age. Furthermore, hemoglobin is related to the rate of oxygen transported in the bloodstream [41].

The concentrations of serum Na and Cl levels were significantly decreased while serum K level was significantly increased in the diarrheic calves compared to healthy calves. The observed changes in the levels of serum Na, Cl and K could be due to excessive water loss with feces which leads to dehydration and impaired cell membrane permeability, in addition to hypovolemia concomitant with the decrease in glomerular filtration rate that plays a key role in the pathogenesis of hyperkalemia [31, 42].

In addition, there was a significant increase in blood urea nitrogen, creatinine, ALT and AST levels, while total protein, serum albumin and glucose concentrations showed significant decreases in the diarrheic calves compared to healthy calves. Blood urea nitrogen and creatinine concentrations were elevated in the diarrheic calves that might be due to deficit in renal blood perfusion thus reducing urine formation and alteration in renal function as hyponatremia, hypochloremia and hyperkalemia as previously reported by Singh et al. [43]. Inflammation of GIT and subsequently pathological affection reflected on the liver [44] might be the cause of elevation of serum AST and ALT in diarrheic calves. On the other hand, serum glucose, Albumin and total protein levels were diminished in the diarrheic calves due to the excretion of those parameters in the intestinal lumen with diarrhea, this was in agreement with Constable et al. [24], Choi et al. [45].

The results of serum NT-proBNP and cTn-I levels were significantly increased, while vitamin D_3_ concentrations showed significant decreases in the diarrheic calves compared to healthy calves. The decrease in Vit D_3_ is attributed to the anorexic phase during the occurrence of diarrhea and the deficiency of Vit D absorption and also the decreased intake with milk and milk replacers. By investigating the relationship between the serum cardiac biomarkers NT-proBNP and cTn-I and the vitamin D_3_ concentrations; the results showed very strong negative correlation as Vit D3 protects against intestinal surface infections and prevents leaky gut syndrome also regulates the inflammatory response and activates immune cells in this process, this coincides with previous reports [20]. Serum cardiac biomarkers such as cardiac troponin I (cTn-I) and NT-proBNP were significantly higher in diarrheic calves compared to clinically healthy calves. Since cTn proteins, which are normally present in blood at very low concentrations or below the limit in detection of most assays, are released into the bloodstream in pericarditis, the large increase in cTn-I and cTn-T concentration in the affected group suggests myocardial cell damage [32]. As well, NT-proBNP levels in the blood are well-established biomarkers for acutely decompensated heart failure. It is rapidly released in the plasma of diseased animal at the acute phases as the cardiomyocytes are stretched mechanically, such ventricular overload or increased wall tension, resulting in an increase in the development of the NT-proBNP hormone precursor (pre-proBNP) [46].

It has been previously described by many researchers the clinical importance of vitamin D_3_ in preventing the inflammatory response in human patients with diarrhea. Thus, studying the relationship between both vitamin D_3_ and inflammation markers will have an impact on explaining the pathophysiology of neonatal calf diarrhea [20, 22]. In addition, vitamin D_3_ protect against infections on the intestinal surface and prevent the development of leaky gut syndrome [20].

## Conclusions

It is concluded that bacterial diarrhea has a severe negative impact on the clinical and hemato-biochemical profile of the neonatal calves with diarrhea. Serum cardiac biomarkers such as cardiac troponin I and NT-proBNP were significantly higher in neonatal calves with diarrhea, and there is negative correlation between cardiac biomarkers and vitamin D levels.

## Data Availability

Not applicable.

## Abbreviations

ALT: Alanine aminotransferase
AST: Aspartate aminotransferase
NT-proBNP: Cardiac N-terminal prohormone B-type natriuretic peptide
cTn-I: Cardiac troponin I
Na: Sodium
K: Potassium
Cl: Chloride
